# On the Implementation of the Artificial Neural Network Approach for Forecasting Different Healthcare Events

**DOI:** 10.3390/diagnostics13071310

**Published:** 2023-03-31

**Authors:** Huda M. Alshanbari, Hasnain Iftikhar, Faridoon Khan, Moeeba Rind, Zubair Ahmad, Abd Al-Aziz Hosni El-Bagoury

**Affiliations:** 1 Department of Mathematical Sciences, College of Science, Princess Nourah bint Abdulrahman University, P.O. Box 84428, Riyadh 11671, Saudi Arabia; 2 Department of Mathematics, City University of Science and Information Technology, Peshawar 25000, Khyber Pakhtunkhwa, Pakistan; 3 Department of Statistics, Quaid-i-Azam University, Islamabad 44000, Pakistan; 4 Department of Economics, Institute of Development Economics, Islamabad 44000, Pakistan; 5 Department of Education, Abasyn University, Peshawar 25000, Khyber Pakhtunkhwa, Pakistan; 6 Department of Psychology, University of Peshawar, Peshawar 25120, Khyber Pakhtunkhwa, Pakistan; 7 Higher Institute of Engineering and Technology, El-Mahala El-Kobra 61111, Egypt

**Keywords:** coronavirus disease 2019, artificial neural network, univariate time series models, forecasting, healthcare phenomena

## Abstract

The rising number of confirmed cases and deaths in Pakistan caused by the coronavirus have caused problems in all areas of the country, not just healthcare. For accurate policy making, it is very important to have accurate and efficient predictions of confirmed cases and death counts. In this article, we use a coronavirus dataset that includes the number of deaths, confirmed cases, and recovered cases to test an artificial neural network model and compare it to different univariate time series models. In contrast to the artificial neural network model, we consider five univariate time series models to predict confirmed cases, deaths count, and recovered cases. The considered models are applied to Pakistan’s daily records of confirmed cases, deaths, and recovered cases from 10 March 2020 to 3 July 2020. Two statistical measures are considered to assess the performances of the models. In addition, a statistical test, namely, the Diebold and Mariano test, is implemented to check the accuracy of the mean errors. The results (mean error and statistical test) show that the artificial neural network model is better suited to predict death and recovered coronavirus cases. In addition, the moving average model outperforms all other confirmed case models, while the autoregressive moving average is the second-best model.

## 1. Introduction

The Coronavirus 2019 (COVID-19) pandemic adversely affected people’s daily lives as well as the economies of countries all over the world. The psychosocial environment was altered significantly because of economic shutdowns, isolation, and social distancing, among other restrictions, and these alterations had a considerable negative impact on countries. Families, young people, and children were particularly hard hit. Due to the requirements for social distancing, there was less opportunity for people to participate in leisure activities, schools and kindergartens were shut down, and fewer opportunities existed for people to interact with one another socially. In contrast, parents were overburdened with work, helping their children with schoolwork, and many of them were working from their homes. In addition to the challenges caused by the economic collapse, unemployment had a substantial influence on the mental health of individuals. In light of the above, it is important to accurately predict COVID-19 data and come up with a plan for the next wave of the pandemic in order to ameliorate the public’s losses. With the help of machine learning tools, we may be able to achieve an accurate forecast for COVID-19 and formulate strategies before confronting the challenges that may arise during the next phase of the pandemic. This, in turn, may lead to a healthy economy for the nation.

Machine learning (ML) is a branch of artificial intelligence that studies and develops ways for computers to learn on their own. ML has been successful in many areas, such as computer vision, detecting fraud, online advertising, automatic driving, and robotics [1]. The success of ML applications in fields, such as treatment, disease diagnosis, patient monitoring, epidemiology, and drug discovery, among others, makes it possible to predict the potential and influence of ML tools in designing and implementing new and better solutions in these areas [2,3]. For instance, Ref. [4] reviewed the significance of using drones, the Internet of Things, artificial intelligence, and blockchain, among other emerging technologies, to combat the pandemic. Similarly, in [5], blockchain is used to propose a method that circumvents the manipulation of information, such as COVID-19 test results.

One of the areas where ML algorithms have implications is the field of health. ML has inspired numerous researchers to approach the study of COVID-19 using a set of ML tools. COVID-19 is an infectious virus that spreads easily and belongs to the family of coronaviruses. The illness produces flu-like signs and symptoms, such as coughing, fever, exhaustion, and shortness of breath. The origin of the virus is still a matter of debate. According to genomic analyses [1], however, this virus is part of the bat- and rodent-hosting coronavirus family and is therefore classified as a member of the Beta-CoV (Corona Virus) genus group. Variants of the virus, including Delta and Omicron, have been responsible for various waves (high peaks) of infections and fatalities across the globe [6]. The Omicron variant, which is considered to be more transmissible but less lethal, was detected in 61.5% of women who reported infections. As of 3 April 2022, more than 491 million confirmed cases and more than 6.1 million deaths had been reported as part of the current COVID-19 pandemic [7,8]. Additionally, it was stated that the pandemic might be over by 2022 and fully under control by 2024 [9]. The scientific community is developing techniques, vaccines, and procedures utilizing various ICT-based technologies and investigating problems to enhance the performance of ML algorithms for survival analysis studies.

Nowadays, time series methods are widely used in statistics, medicine, health science, financial mathematics, pattern recognition, communications engineering, astronomy, and many other fields of applied science and economics that involve time-based measurements. Time series models are an important part of forecasting in the medical field because they have their own unique properties [10]. For example, Ref. [11] used the autoregressive integrated moving average model (ARIMA) to predict the number of COVID-19 deaths and recoveries in Pakistan. The authors in [12] predict the future spread of COVID-19 by exploiting lead–lag effects identified in different countries. Specifically, they first determine the past relationships between nations with the help of dynamic time loops. The method presented applies to confirmed coronavirus cases from 1 January 2020 to 28 March 2020. The results show that China leads all other countries in the range of 29 days for South Korea and 44 days for the United States. Ref. [13] forecasted the epidemic peak of COVID-19 in Turkey, Brazil, and South Africa using an age-structured SEIR system. Some researchers predicted the continuation of COVID-19 using the exponential smoothing method. For example, Ref. [14] explored the development of informational efficacy in cryptocurrency and international stock markets before and during the pandemic caused by COVID-19. They found that the crypto markets were more unstable during the COVID-19 pandemic than international stock markets. Thus, investing in digital assets during pandemic times might be riskier.

Few authors used machine learning models for forecasting COVID-19 [15,16,17,18]. The work [19] investigated the spread of COVID-19 using the case of Malaysia and scrutinized its linkage with some external factors, e.g., inadequate medical resources and incorrect diagnosis problems. They used an epidemiological model and a dynamical systems technique and observed that this might misrepresent the evaluation of the severity of COVID-19 under complexities. Ref. [20] discusses a comprehensive review of studies applying deep learning (DL) models for the diagnosis of COVID-19 and lung segmentation. In addition, an overview of work on predicting coronavirus prevalence in different parts of the world with DL is presented. Finally, challenges in detecting COVID-19 using DL techniques and directions for future research are discussed. Based on the spreading behavior of COVID-19 in the population, Ref. [21] estimated three novel quarantine epidemic models. They found that isolation at home and quarantine in hospitals are the two most effective control strategies under the current circumstances when the disease has no known available treatment. In the work [22], using positive cases over 50 days of disease progression for Pakistan, the authors analyzed the graphical trend and forecasted the behavior of disease progression through exponential growth for the next 30 days. They assumed different possible trajectories and projected an estimated 20k–456k positive cases within 80 days of disease spread in Pakistan.

Yaqoob et al. [23] introduced two-dimensional reduction procedures, feature extraction, and feature selection, as well as a systematic comparison of various dimension reduction procedures for the analysis of high-dimensional gene expression biomedical data. This paper can assist researchers in selecting the most efficient algorithm for cancer classification and prognosis in order to analyze high-dimensional biomedical data satisfactorily.

The proposed technique and support vector classification model beat the other models in terms of accuracy, whereas deep learning along with the proposed optimization approach beat the random forest model with 99.71% versus 98.33% performance [24]. Sagu et al. [25] introduced new dual metaheuristic optimization algorithms for adjusting the weights of DL models. Using DL may assist in the unmasking and prevention of cyberattacks. In addition, dual hybrid DL classifiers, i.e., convolutional neural network + deep belief network and bidirectional long short-term memory + gated recurrent network, were devised and tuned utilizing the previously proposed optimization algorithms, resulting in improved model accuracy. Iftikhar et al. [26] conducted a study using the chronic kidney disease dataset and attempted a comparison of various machine-learning techniques. Results show that in all three scenarios, the SVM-LAP model is superior to rival approaches.

### 1.1. An Overview of the COVID-19 Pandemic

COVID-19 is an infectious disease that is spreading rapidly in populated areas. The World Health Organization declared COVID-19 a global pandemic that has affected at least 99% of the countries in the world, first identified in the city of Wuhan, Hubei Province of China [27]. The humanitarian costs of the COVID-19 outbreak have been rising since 31 December 2019, as it affected more than 10,710,005 people and resulted in a death count of 517,877 through 3 July 2020 globally [28]. The countries that share borders with Pakistan were infected by COVID-19, including Iran and China, which were the major influencing factors for Pakistanis. The first two cases were confirmed on 26 February 2020 in Islamabad and Karachi [29]. Due to the weak healthcare system of the country, many people were affected, and careless public attitudes and mega shopping made the coming days worst. On 13 March, the government of Pakistan imposed a complete lockdown on the whole country. In the continuation of the lockdown, authorities took the initial steps to reduce the spread of the virus: canceling conferences to disrupt supply chains, imposing travel restrictions, closing borders, canceling flights, and closing shopping malls, schools, colleges, and universities, To raise awareness, different TV programs, commercials, and advertisements were organized, and face masks and sanitizers were used by everyone [30]. After the partial lifting of the lockdown on 15 April 2020 and further relaxation on 12 May 2020, the number of cases increased dramatically. During May, more than fifty thousand new cases were reported. The rise did not stop there, and the month of June proved to be worse. The total number of cases and confirmed deaths in the country as of 3 July 2020, was 198,883 and 4035, respectively. Sindh reported the highest number of cases, which was 76,318, followed by Punjab at 72,880. At the same time, Punjab had the highest number of deaths in the country, with 1656, followed by Sindh with 1205 [31]. A continuous struggle is required to reduce the spread of COVID-19 so that the healthcare sector can deal with COVID-19 patients in the future [32].

Due to the mutated nature of the virus, the situation has become graver as little is known about the cure, and the probable timeline of this disease remains highly uncertain. Hence, forecasting for the short-term is immensely important in finding a clue for predicting the flattening of curves and the revival of routine social and economic life [33,34]. Statistical models using evidence from real-world data can help predict the location, timing, and size of outbreaks, allowing governments to allocate resources more effectively, conduct scenario and signal analysis, and determine policy approaches. Epidemiological tools are applied to limit the scope and spread of outbreaks; however, these approaches are sensitive to the underlying assumptions, and their impact varies [35,36]. It is essential to ensure oversight by checking assumptions in modeling and ensuring the veracity, reliability, and accountabilities these tools use to address bias and other potential harms. This work attempts to look at the projections for COVID-19 infections in Pakistan using several univariate time series methods along with an artificial neural network (ANN) approach.

### 1.2. Contribution of the Study

This study contributes to the literature on forecasting COVID-19 in several ways: The study considers two kinds of tools: parametric and non-parametric, including an artificial neural network. In a similar way, our study uses three kinds of data on COVID-19, i.e., confirmed cases, confirmed deaths, and recovered cases in Pakistan. Third, the study compares parametric and non-parametric techniques, including ANN, statistically as well as graphically and selects the best technique. The best technique is then used for future forecasting of the confirmed, deceased, and recovered cases.

### 1.3. Organization of the Study

The rest of the article is organized as follows: Section 2 describes the materials and methods. Section 3 discusses training, testing, and prediction model results and discusses future forecasts. Finally, Section 4 contains conclusions, limitations, and future directions.

## 2. Materials and Methods

Five different univariate time series models, including autoregressive (AR), moving average (MA), autoregressive moving average (ARMA), nonparametric autoregressive (NPAR), and simple exponential smoothing (SES), as well as one machine learning model, an artificial neural network model, are employed in this study. These models are described in the following subsections:

### 2.1. Autoregressive Process

A linear AR process describes a linear function of the previous n observations of $M_{\left(t\right)}$, which is defined as:

(1) $$M_{t}=\alpha +\gamma_{1}M_{t-1}+\gamma_{2}M_{t-2}+\ldots +\gamma_{n}M_{t-n}+e_{t},$$

where α and $\gamma_{i}\left(i=1,2,\cdots ,n\right)$ are the intercept and slope coefficients of the underlying AR process, and $e_{t}$ is the disturbance term.

### 2.2. Moving Average Model

The MA model primarily removes the periodic fluctuations in the time series data, for example, fluctuations due to seasonality. The MA model can be written mathematically as:

(2) $$M_{t}=\alpha +e_{t}+\phi_{1}e_{t-1}+\phi_{2}e_{t-2}+\cdots +\phi_{s}e_{t-s},$$

where α indicate the constant (intercept), $e_{j}(j=1,2,\cdots ,s)$ are parameters of the MA model, and $e_{t}$ is a white process. The values s reveal the order of the MA process.

### 2.3. Nonparametric Autoregressive Model

The additive nonparametric counterpart of the AR process leads to the additive model. The association between $M_{t}$ and its previous lags has a nonlinear relationship, which is described as:

(3) $$M_{t}=g_{1}\left(M_{t-1}\right)+g_{2}\left(M_{t-2}\right)+\ldots +g_{k}\left(M_{t-n}\right)+e_{t},$$

where $g_{i}\left(i=1,2,\cdots ,k\right)$ shows the smoothing function and describes the association between $M_{t}$ and its previous values, and further $g_{i}$ is denoted by cubic regression splines. As in the case of the parametric form, we utilized 2 lags while estimating NPAR.

### 2.4. Autoregressive Moving Average Model

The ARMA model is defined as the response variable $M_{t}$ regressed on the previous n lags with residuals (errors) as well. Mathematically,

(4) $$M_{t}=\alpha +\gamma_{1}M_{t-1}+\gamma_{2}M_{t-2}+\cdots +\gamma_{r}M_{t-n}+e_{t}+\phi_{1}e_{t-1}+\phi_{2}e_{t-2}+\ldots +\phi_{m}e_{t-m},$$

where α denotes the intercept, $\gamma_{i}\left(i=1,2,\cdots ,n\right)$ and $\phi_{k}\left(k=1,2,\cdots ,m\right)$ are the parameters of AR and MA processes, respectively, and $e_{t}$ is a Gaussian white noise series with mean zero and variance $\sigma_{e}^{2}$. The ARMA model order selection is established by inspecting the correlograms, i.e., partial and auto-correlation functions. In our case, we fit an ARMA (1, 1) model to each series $M_{t}$.

### 2.5. Simple Exponential Smoothing Model

The SES model for forecasting allows the researchers to smooth the time series data and then use it for out-of-sample forecasting. The SES model is applicable when the data are stationary, such as with no trend and no seasonal pattern. However, the data at the level change gradually over time.

(5) $${\hat{M}}_{t+1,k}=\gamma_{1}M_{t}+\left(1-\gamma_{1}\right)\;{\hat{M}}_{t,k},$$

where $\gamma_{1}$ is the smoothing constant, $M_{t}$ is the actual series, ${\hat{M}}_{t,k}$ is the forecasted value of the underlying series for the period *t*, and ${\hat{M}}_{t+1,k}$ is the forecasted value for the period t+1. This method assigns the weights in such a way that moving back from the current value, the weights exponentially decrease.

### 2.6. Artificial Neural Network

Artificial neural networks (ANNs) are adaptive computing frameworks for modeling a wide range of nonlinear problems. Unlike other nonlinear models, ANNs can approximate a wide variety of functions more accurately. This is the main advantage of ANN. Their efficiency is based on the parallel processing of data information. The modeling process does not provide any knowledge of the model’s geometry. Instead, data properties play a large role in determining network models. One of the most popular types of artificial neural networks for time series modeling and forecasting is the multilayer perceptron with hidden layers, which is especially commonly used. Three layers of simple processing units are connected with circular connections to form a network that defines the model. The following equation describes the relationship between the output ($M_{t}$) and the inputs ($M_{\left(t-1\right)},M_{\left(t-2\right)}\ldots ,M_{\left(t-n\right)}$)

(6) $$M_{t}=\alpha +\Sigma_{k=1}^{z}g_{k}\varphi \left(g_{0k}+\Sigma_{j=1}^{n}M_{t-j}\right).$$

In the above formula given in Equation (6), the model parameters are indicated by $g_{\left(j,k\right)}$ (j = 0, 1, 2, …, n; k = 1, 2, …, z) and $g_{k}$ (j = 0, 1, 2, …, z) and are often known as connection weights; n shows the length of input nodes; and z shows the length of hidden nodes.

### 2.7. Performance Measures

To check the effectiveness of the forecasting models in the literature, many researchers used different accuracy measures and statistical tests [37,38]. However, in this work, for model evaluation, first, we used two accuracy mean errors; one absolute mean error and one relative mean error, namely mean absolute error (MAE) and root mean square error (RMSE). The mathematical formula for accuracy mean errors are given by

$$MAE=\mathrm{Mean}\;(|M_{t}-{\hat{M}}_{t}|),$$

and

$$RMSE=\sqrt{\mathrm{Mean}{\left(M_{t}-{\hat{M}}_{t}\right)}^{2}}\;\;,$$

where $M_{t}\;$ = observed, and ${\hat{M}}_{t}\;$ = predicted values for *t*-th day (*t*: 1, 2, ⋯, 45).

Second, to assess the significance of the differences in the forecasting performance of the models, the Diebold and Mariano test was performed [39]. The DM test is a widely used statistical test for comparing forecasts obtained from different models [40,41]. To understand it, consider two forecasts, ${\hat{M}}_{1t}$ and ${\hat{M}}_{2t}$, that are available for the time series $M_{t}$ for t=1,⋯,T. The associated forecast errors are $e_{1t}=M_{t}-{\hat{M}}_{1t}$ and $e_{2t}=M_{t}-{\hat{M}}_{2t}$. Let the loss associated with forecast error ${\left\{e_{it}\right\}}_{i=1}^{2}$ be $L(e_{it})$. For example, time *t* absolute loss would be $L\left(e_{it}\right)=\left|e_{it}\right|$. The loss differential between Forecasts 1 and 2 for time *t* is then $w_{t}=L\left(e_{1t}\right)-L\left(e_{2t}\right)$. The null hypothesis of equal forecast accuracy for two forecasts is $E[w_{t}]=0$. The DM test requires that the loss differential be covariance stationary, i.e.,

$$E[w_{t}]=\mu ,\;\forall \;t,$$

$$\mathrm{cov}\left(w_{t}-w_{t-\tau }\right)=\gamma \left(\tau \right),\;\forall \;t,$$

and

$$\mathrm{var}\left(w_{t}\right)=\sigma_{w},\;0<\sigma_{w}<\infty .$$

Under these assumptions, the DM test of equal forecast accuracy is

$$\mathrm{DM}=\frac{\bar{w}}{{\hat{\sigma }}_{\bar{w}}}\overset{d}{\to }N\left(0,1\right),$$

where $\bar{w}=\frac{1}{T}\sum_{t=1}^{T}w_{t}$ is the sample mean loss differential, and ${\hat{\sigma }}_{\bar{w}}$ is a consistent standard error estimate of $w_{t}$.

**Figure 1:**
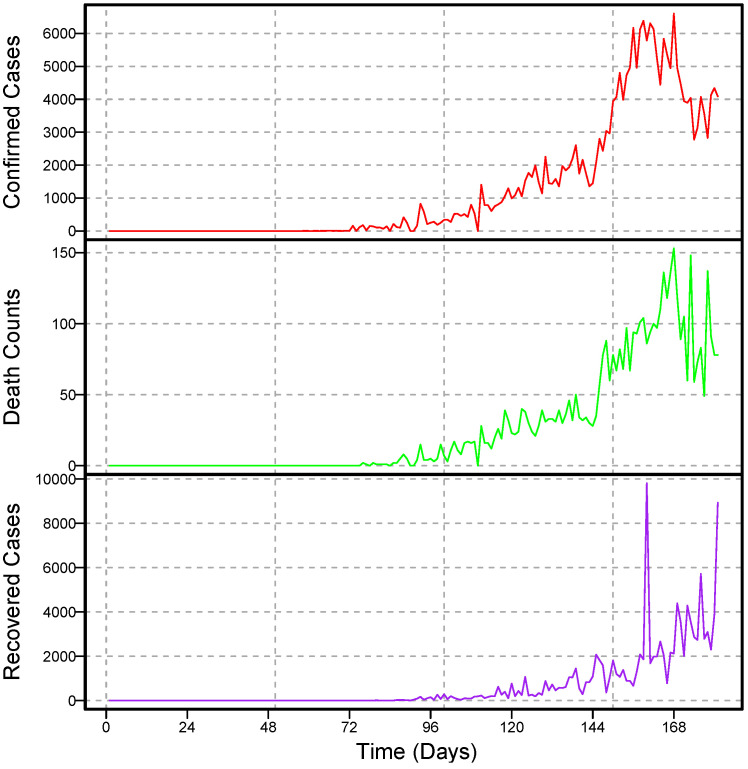
Pakistan COVID-19: daily confirmed cases (1st panel), death counts (2nd panel), and recovered cases (3rd panel) over the period of 28 February to 3 July 2020.

**Figure 2:**
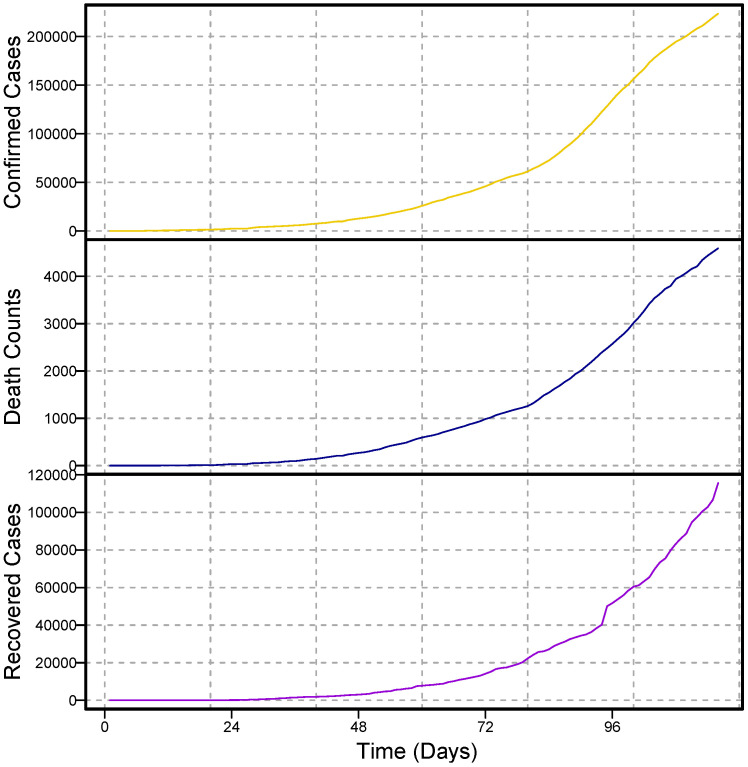
Pakistan COVID-19: cumulative confirmed cases (1st panel), death counts (2nd panel), and recovered cases (3rd panel) over the period of 28 February to 3 July 2020.

**Figure 3:**
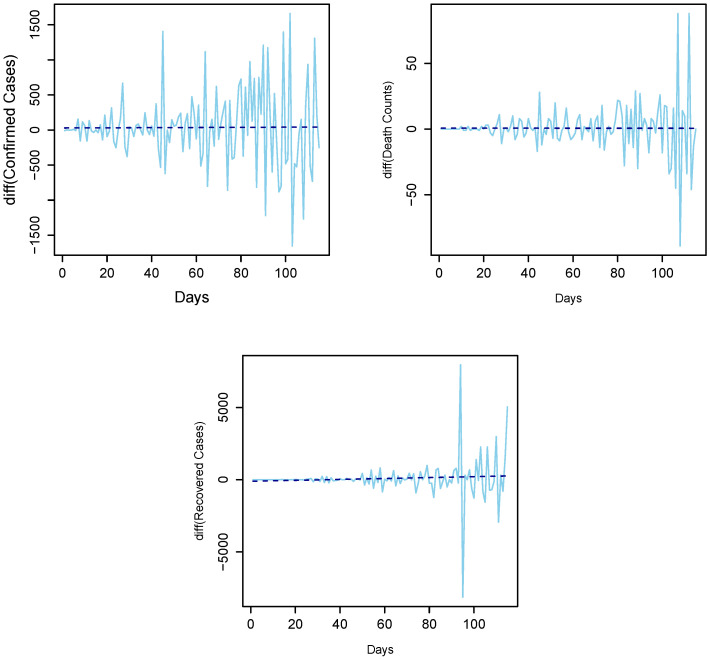
Differenced series: 1st order difference for confirmed cases (**top left**), death counts (**top right**), and recovered cases (**bottom center**).

For modeling, one of the most important things to assume about time series data is that it is stationary. A stationary process is one in which the mean, the variance, and the structure of the autocorrelation do not change over time. If the underlying series is nonstationary, it should be converted to stationary. In the literature, different techniques are used to achieve stationarity, for example, taking the natural log and differencing the series or box-cox transformation [42]. In this work, the daily COVID-19 confirmed cases, deaths, and recovery time series are plotted in Figure 1 for daily and Figure 2 for cumulative cases. As seen, all three time series have an upward increasing linear trend, which shows that the series are non-stationary, hence the need to make them stationary using the differencing method. In addition, to check the unit root issue of the aforementioned series statistically, we apply the augmented Dickey–Fuller test. The results are listed in Table 1, which suggests that all three series are nonstationary at the level. However, taking the first-order difference into account, the series turned out to be stationary. The first order differencing series of daily confirmed cases, deaths, and recovered cases is depicted in Figure 3, which ensures stationarity.

## 3. Experimental Results and Discussion

The study used daily data from confirmed COVID-19 cases, deaths, and recovered cases from Pakistan. The dataset was obtained from the World Health Organization; each series ranges from 10 March 2020 to 3 July 2020. The descriptive statistics of the considered datasets are given in Table 2. For practical and rational modeling through time series models, at least 30 observations were required [43]. To do this, approximately 116 data points from each series were considered. The complete dataset covers 116 days, of which 10 March 2020 to 19 May 2020 (71 days) were used for model training, and 21 May to 3 July 2020 (45 days) were used for one-day-ahead post sample (testing) predictions.

**Figure 4:**
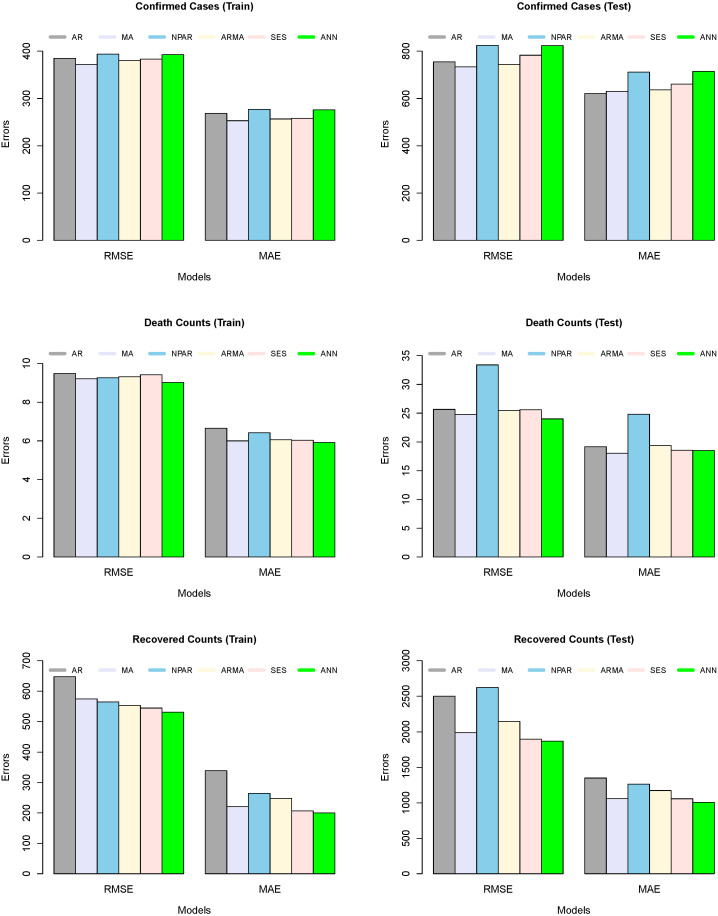
Barplot: RMSE and MAE for confirmed cases, deaths, and recovered cases; **Model Estimation/Train (left column)**, **Out-of-Sample/Test (2nd column)** for all models.

We used two accuracy measures (MAE and RMSE) to figure out which model for each series is the best. The results of these accuracy measures are shown in Table 3 and Table 4. Table 3 shows the numerical description of the trained model’s accuracy mean errors for the all-considered model, such as five time series models and a machine learning model. On the other hand, the table presented a numerical description of the tested model’s accuracy and mean errors for all considered models. From the output of both Table 3 and Table 4, we can observe that the MA model produced low forecast errors, in contrast to all other competitors for confirmed predictions. The RMSE and MAE values for the MA model are 733.92 and 629.95, respectively, for confirmed cases. However, the ARMA model remains a good competitor. In the case of predicted death counts and recovered patients due to COVID-19, the ANN algorithm shows better results than the rest of the models, while the MA and SES models are the second- and third-best models, respectively. In addition, a graphical analysis of the RMSE and MAE values for confirmed cases, death counts, and recovered patients is plotted in Figure 4. However, Figure 4 (left column) shows the graphical representation of the trained model’s accuracy mean errors for all considered models. On the other side, Figure 4 (right column) shows the graphical representation of the test model’s accuracy mean errors for all considered models. The superiority of the MA (confirmed cases) and ANN (death counts and recovered patients) models can be seen in both training and testing exercises.

**Figure 5:**
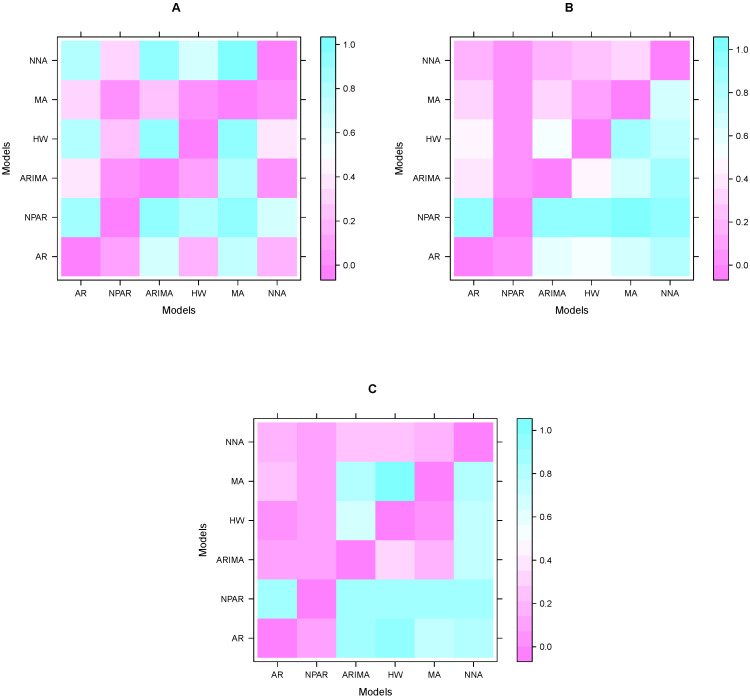
Results (*p*-value) of the DM test for confirmed cases (**A**), death counts (**B**), and recovered cases (**C**).

Once the performance of models is calculated by accuracy mean errors. The next step is to assess the dominance of these results. For this purpose, many researchers in the literature performed the Diebold and Mariano test (DM). In this work, we performed a DM test on each pair of models to verify the superiority of the model results (performance indicators) shown in Table 4. The DM test results (*p*-values) for confirmed cases are shown in Table 5. The null hypothesis is displayed as a predictor in contrast to the alternative where all entries in the table are *p*, and the accuracy of the column/row predictors are more accurate than the column/row predictors of the hypothesis system. This table shows that among all the models in Table 4 (confirmed cases), the MA model is statistically superior to the other models at the 5% significance level. The DM test results (*p*-values) for the number of deaths are shown in Table 6. This table confirms that among all the models in Table 4 (death counts), the ANN and MA models are statistically superior to the other at 5% significance level models. In addition, DM test results (*p*-values) for recovered cases are shown in Table 7. The results in these tables show that among all the models in Table 4 (recovered cases), the ANN and SES models are statistically superior to the other models at the 5% significance level. On the other hand, the graphic representation of these results (*p*-values) is presented in Figure 5. The sky blue color in Figure 5, is close to one, which means that the difference between the two models is significant; in contrast, the purple color indicates that the two models are not statistically significant at the 5% significance level. Thus, the superiority of the models in each case is easily seen in the figures. Therefore, from the descriptive statistics, graphical interpretation, and a statistical test, the superiority of the models in each case is confirmed.

**Figure 6:**
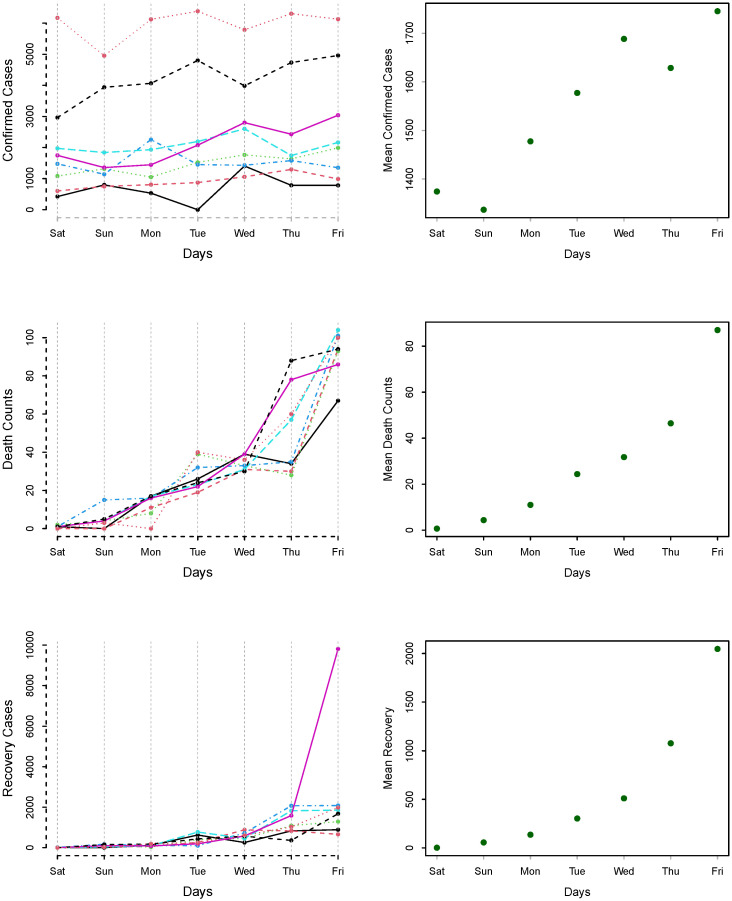
Weekly COVID-19 cases: day-specific confirmed cases, deaths, and recovered cases; (**left column**) and mean day-specific (**right column**) for the period of 21 March to 19 June 2020.

The day-specific confirmed cases, deaths, and recovered counts are plotted in Figure 6 for 21 March to 19 June 2020. Figure 6 (left column) shows that there is variation among the different weeks, while in Figure 6 (right-column), the mean of days are plotted for confirmed cases, deaths, and recovered cases. We can see an increasing pattern from Saturday to Friday, which shows the effect of working and non-working days.

**Figure 7:**
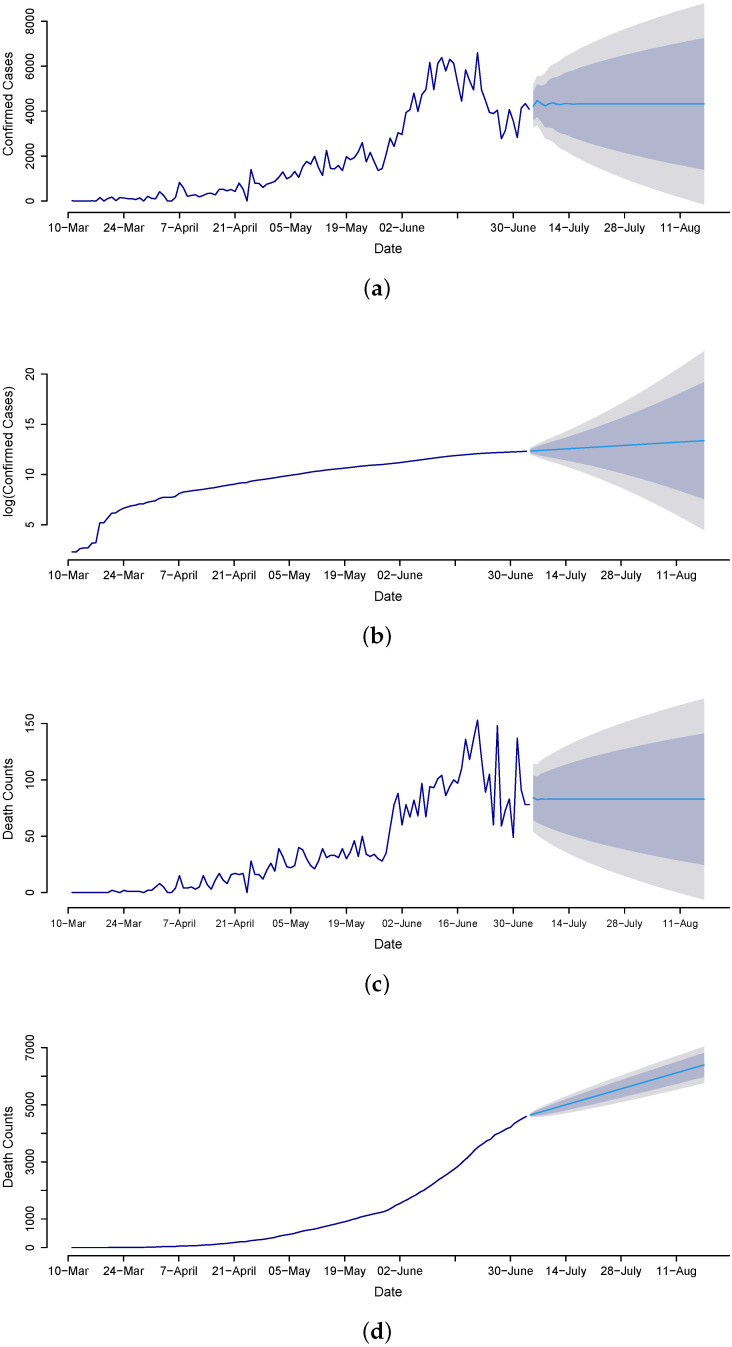

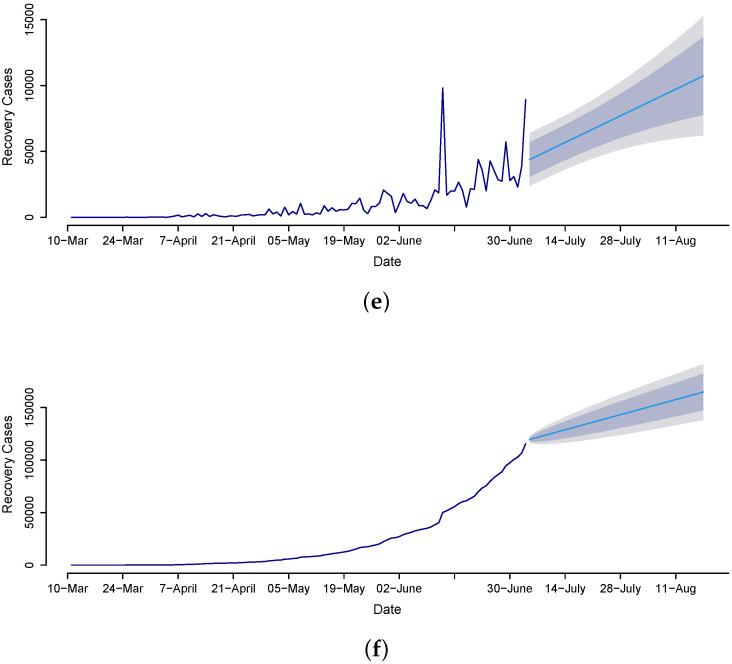
Forecasts for COVID-19: Confirmed daily and cumulative cases using the MA model (**a**,**b**), daily and cumulative deaths using the ANN model (**c**,**d**), and daily and cumulative recovered cases using the ANN model (**e**,**f**) for the period 3 July to 14 August 2020.

Once the best models are assessed through the out-of-sample mean errors (RMSE, MAE), a statistical test (DM test), and graphical analysis, we proceed to the future forecasting of confirmed cases, death counts, and recovered cases. Therefore, we implement the MA model for confirmed cases and the ANN model for the death count and recovered cases to forecast from 4 July to 14 August 2020, for the daily and cumulative number of cases. The forecasted values are presented in Figure 7, clearly revealing that death counts and recovered cases are monotonically increasing while confirmed cases are not. The confirmed cases on 14 August 2020 are expected to be 7325, and the cumulative is 413,639. The death counts during late August are expected to be 121, and the cumulative counts are 9279. The recovered cases are 10,730, and the cumulative count is 455,661. Overall, the results suggest that the trend in confirmed cases gradually decreased over time, which is the outcome of the earlier steps that the government imposed, such as canceling conferences to disrupt supply chains; imposing travel restrictions; closing borders; canceling flights; closing workplaces; closing shopping malls, schools, colleges, and universities; and raising awareness through different TV programs, commercials, and advertisements, as well as having everyone use face masks and sanitizers.

## 4. Conclusions

The main purpose of this work was to forecast confirmed cases, death counts, and recovered cases of coronavirus in Pakistan using a machine learning model and five different univariate time series models, such as autoregressive, moving average, autoregressive moving average, nonparametric autoregressive, and simple exponential smoothing models. These models were applied to Pakistan’s daily records of confirmed cases, death counts, and recovered cases from 10 March 2020 to 3 July 2020. To evaluate the performances of the fitted models, a statistical test and two mean errors were considered. Experimental results showed that the ANN model outperformed the time series models considered in this study. Using the recovered cases, for the ANN model, the values of RMSE and MAE were 1870.07 and 1006.91, respectively. Using the death cases, for the ANN model, the values of RMSE and MAE were 24.00 and 17.89, respectively. On the other hand, using the confirmed cases, the MA model outperformed the ANN and other time series models. Using the confirmed cases, for the MA model, the values of RMSE and MAE were 733.92 and 629.95, respectively. Furthermore, the performances of the fitted models were assessed using the Diebold and Mariano test. The Diebold and Mariano’s test results (*p*-values) showed that among all models (confirmed cases), the MA model was statistically superior to the other models at the 5% significance level. On the other hand, for predicting mortality and recovery cases, the ANN model was statistically superior to the rest of all models at the 5% significance level. Based on the best-selected models, we forecasted confirmed cases and death counts from 4 July to 14 August 2020, which will be helpful for the decision making of public healthcare and other sectors in Pakistan.

This work only compares univariate models; no multivariate time series models are used. In the future, considering the covariates that affect COVID-19 can improve the forecasting performance of the models. In addition, machine learning models, such as random forest and support vector regression, can be used to obtain more accurate and efficient predictions in the future.

## Figures and Tables

**Table 1:** Augmented Dickey-Fuller test statistics.

At Level		At First Difference	
Variables	Constant with Trend	Constant with Trend	Conclusion
Cases	−1.806	−10.447	I (1)
Deaths	−1.022	−7.470	I (1)
Recoveries	−0.095	−6.348	I (1)

**Table 2:** The descriptive statistics of the considered datasets.

Measures	CFC	CFD	CFR	Cases	Deaths	Recoveries
Mean	35,905	727.4	13,877	1235	25.38	638.7
STD	60,709.8	1220.4	26,118.1	1811.8	37.48	1356.5
Kurtosis	2.07	2.17	3.99	0.99	1.55	19.47
Skewness	1.81	1.81	2.19	1.47	1.57	3.87

Note: cumulative confirmed cases (CFC), cumulative deaths counts (CFD), and cumulative recovered cases (CFR).

**Table 3:** **Model Estimation/Train**: One-day-ahead RMSE and MAE for confirmed cases, deaths, and recovered cases for all models.

Model Estimation/Train						
	**Confirmed**		**Deaths**		**Recoveries**	
**MODELS**	**RMSE**	**MAE**	**RMSE**	**MAE**	**RMSE**	**MAE**
**AR**	385.02	268.58	9.48	6.65	647.34	338.87
**MA**	**371.74**	**252.98**	9.21	6.00	574.14	220.60
**NPAR**	393.85	277.07	9.26	6.42	564.36	264.02
**ARMA**	380.69	256.66	9.31	6.06	552.89	247.70
**SES**	383.17	257.93	9.42	6.03	544.18	206.87
**ANN**	392.84	276.07	**9.02**	**5.92**	**530.86**	**200.07**

**Table 4:** **Out-of-Sample/Test**: One-day-ahead RMSE and MAE for confirmed cases, deaths, and recovered cases for all models.

Out-of-Sample/Test						
	**Confirmed**		**Deaths**		**Recoveries**	
**MODELS**	**RMSE**	**MAE**	**RMSE**	**MAE**	**RMSE**	**MAE**
**AR**	755.07	620.95	25.65	19.17	2500.20	1349.63
**MA**	**733.92**	**629.95**	24.78	18.02	1987.75	1059.44
**NPAR**	824.53	711.87	33.39	24.79	2623.00	1264.31
**ARMA**	743.24	636.31	25.46	19.36	2143.37	1173.68
**SES**	782.89	661.09	25.60	18.55	1897.32	1057.09
**ANN**	823.84	714.41	**24.00**	**17.89**	**1870.07**	**1006.91**

**Table 5:** Results (*p*-value) of the DM test for all the considered models using the confirmed cases.

Confirmed Cases						
**Models**	**AR**	**NPAR**	**ARIMA**	**SES**	**MA**	**ANN**
**AR**	0.00	0.89	0.36	0.82	0.30	0.83
**NPAR**	0.11	0.00	0.07	0.24	0.04	0.41
**ARIMA**	0.64	0.93	0.00	0.92	0.23	0.95
**SES**	0.18	0.76	0.08	0.00	0.05	0.70
**MA**	0.70	0.96	0.77	0.95	0.00	0.97
**ANN**	0.17	0.59	0.05	0.30	0.03	0.00

**Table 6:** Results (*p*-value) of the DM test for all the considered models using the death counts.

Death Counts						
**Models**	**AR**	**NPAR**	**ARIMA**	**SES**	**MA**	**ANN**
**AR**	0.00	0.97	0.40	0.49	0.30	0.21
**NPAR**	0.03	0.00	0.02	0.01	0.01	0.02
**ARIMA**	0.60	0.98	0.00	0.54	0.30	0.16
**SES**	0.51	0.99	0.46	0.00	0.10	0.24
**MA**	0.70	0.99	0.70	0.90	0.00	0.36
**ANN**	0.79	0.98	0.84	0.76	0.64	0.00

**Table 7:** Results (*p*-value) of the DM test for all the considered models using the recovered cases.

Recovered Cases						
**Models**	**AR**	**NPAR**	**ARIMA**	**SES**	**MA**	**ANN**
**AR**	0.00	0.88	0.13	0.05	0.27	0.18
**NPAR**	0.12	0.00	0.12	0.10	0.12	0.13
**ARIMA**	0.87	0.88	0.00	0.65	0.82	0.23
**SES**	0.95	0.90	0.35	0.00	0.99	0.28
**MA**	0.73	0.88	0.18	0.01	0.00	0.20
**ANN**	0.82	0.87	0.77	0.72	0.80	0.00

## Data Availability

The data sets are available from the corresponding author upon reasonable request.
